# Retraction Note: Curcumin inhibits the migration of osteoclast precursors and osteoclastogenesis by repressing CCL3 production

**DOI:** 10.1186/s12906-024-04647-3

**Published:** 2024-09-27

**Authors:** Zhengeng Liang, Yan Xue, Tao Wang, Qi Xie, Jiafu Lin, Yu Wang

**Affiliations:** 1 Department of Stomatology, Hainan General Hospital, Hainan Affiliated Hospital of Hainan Medical University, Haikou, 570000 China; 2 Fujian Health College, Fuzhou, Fujian 350000 China; 3 Department of Orthopaedics, Chifeng Municipal Hospital, Chifeng, Inner Mongolia 024000 China; 4 Chifeng Clinical Medical School of Inner Mongolia Medical University, Chifeng, Inner Mongolia 024000 China

**Retraction Note: BMC Complementary Medicine and Therapies (2020) 20:234**

10.1186/s12906-020-03014-2

RETRACTION NOTE.

The Editor has retracted this article. After publication, concerns were raised regarding the M + R + CCL3 CUR image in Fig. 3a, which appears to overlap with M + R + LV-Cont PBS in Fig. 4a. The authors have requested a correction and provided the underlying raw data to address this. However, further checks by the publisher have found a number of areas of high similarity between raw data images representing different treatment groups.

The Editor therefore no longer has confidence in the presented data.

None of the authors have responded to any correspondence from the editor or publisher about this retraction notice.

